# Ultrasound Estimates of Visceral and Subcutaneous-Abdominal Adipose Tissues in Infancy

**DOI:** 10.1155/2013/951954

**Published:** 2013-04-17

**Authors:** Emanuella De Lucia Rolfe, Neena Modi, Sabita Uthaya, Ieuan A. Hughes, David B. Dunger, Carlo Acerini, Ronald P. Stolk, Ken K. Ong

**Affiliations:** ^1^MRC Epidemiology Unit, Institute of Metabolic Science, Addenbrooke's Hospital, P.O. Box 285, Cambridge CB2 0QQ, UK; ^2^Department of Epidemiology, Groningen University, 9700 RB Groningen, The Netherlands; ^3^Section of Neonatal Medicine, Department of Medicine Chelsea and Westminster Hospital Campus, Imperial College London, London SW10 9NH, UK; ^4^Department of Paediatrics, University of Cambridge, Addenbrooke's Hospital, P.O. Box 116, Cambridge CB2 0QQ, UK

## Abstract

Other imaging techniques to quantify internal-abdominal adiposity (IA-AT) and subcutaneous-abdominal adiposity (SCA-AT) are frequently impractical in infants. The aim of this study was twofold: (a) to validate ultrasound (US) visceral and subcutaneous-abdominal depths in assessing IA-AT and SCA-AT from MRI as the reference method in infants and (b) to analyze the association between US abdominal adiposity and anthropometric measures at ages 3 months and 12 months. Twenty-two infants underwent MRI and US measures of abdominal adiposity. Abdominal US parameters and anthropometric variables were assessed in the Cambridge Baby Growth Study (CBGS), *n* = 487 infants (23 girls) at age 3 months and *n* = 495 infants (237 girls) at 12 months. US visceral and subcutaneous-abdominal depths correlated with MRI quantified IA-AT (*r* = 0.48, *P* < 0.05) and SCA-AT (*r* = 0.71, *P* < 0.001) volumes, respectively. In CBGS, mean US-visceral depths increased by ~20 % between ages 3 and 12 months (*P* < 0.0001) and at both ages were lower in infants breast-fed at 3 months than in other infants. US-visceral depths at both 3 and 12 months were *inversely* related to skinfold thickness at birth (*P* = 0.03 and *P* = 0.009 at 3 and 12 months, resp.; adjusted for current skinfold thickness). In contrast, US-subcutaneous-abdominal depth at 3 months was *positively* related to skinfold thickness at birth (*P* = 0.004). US measures can rank infants with higher or lower IA-AT and SCA-AT. Contrasting patterns of association with visceral and subcutaneous-abdominal adiposities indicate that they may be differentially regulated in infancy.

## 1. Introduction

Childhood obesity has become a major public health issue and its prevalence is increasing worldwide [1–3]. More important than BMI, or overall adiposity, greater abdominal distribution of adiposity is associated with insulin resistance, dyslipidemia, hyperinsulinemia, and hypertension [4–6]. In obese children, greater internal-abdominal adiposity (IA-AT), also known as visceral fat, is associated with less favourable metabolic profiles [7, 8]. In addition, subcutaneous-abdominal adipose tissue (SCA-AT) is also associated with insulin resistance and metabolic disorders in some studies [9, 10]. 

Several epidemiological studies have reported that early life factors, such as impaired fetal growth or excess postnatal weight gain, are associated with later obesity and related comorbidities [11–15]. Growth in fetal life as well as in infancy has been associated with subsequent abdominal adipose tissue accumulation [11, 16]. However, those studies used indirect measures of abdominal adiposity, such as skinfold thickness and waist-hip ratio, and therefore could not distinguish between IA-AT and SCA-AT compartments. Computed tomography (CT) and magnetic resonance imaging (MRI) are considered the gold standards for the assessment of IA-AT and SCA-AT. However, their use is limited in research studies in young children due to high sensitivity to movement artefacts, exposure to ionising radiation (CT only) and need for expensive equipment and specialist technicians [17, 18]. MRI has previously been used to quantify IA-AT and SCA-AT at birth [19]. However, between ages 3-4 months and around 5-6 years, MRI is not feasible in research studies as sedation or even general anaesthesia is required. Therefore research studies in infants usually use anthropometry to evaluate adiposity. However, these measures do not differentiate between IA-AT and SCA-AT.

Ultrasound (US) has been assessed as a noninvasive estimate of IA-AT and SCA-AT. US-visceral depth and US abdominal-subcutaneous depth have been shown to be reliable and reproducible estimates of IA-AT and SCA-AT, respectively, when compared to CT or MRI in adults and in adolescents [20–24]. However, its validity has not been studied in infants. We therefore tested the validity of US-visceral depth and US-abdominal subcutaneous depth by comparison to MRI measures of IA-AT and SCA-AT volumes in newborn infants. In addition, we used this technique to analyze the cross-sectional and prospective associations between US abdominal adiposity and anthropometric variables in the first year of life of a large birth cohort study.

## 2. Population and Methods

### 2.1. Validation Study

The validation study was carried out in a convenience sample of 22 healthy term singleton newborn infants (10 boys and 12 girls). Mothers and babies were recruited from the Neonatal Unit and postnatal wards of the Chelsea and Westminster Hospital, London, UK, between 2008 and 2009 and attended the Robert steiner MRI Unit, Hammersmith Hospital, London, UK. This study was approved by the Hammersmith and Queen Charlotte's & Chelsea Hospital research ethics committee. Written parental consent was obtained prior to the participants' visit.

### 2.2. Cambridge Baby Growth Study (CBGS)

Details of the study have been described elsewhere [25]. Briefly, mothers were recruited from the Rosie Maternity Hospital, Cambridge, UK, between 2001 and 2009 at their first antenatal clinic by trained paediatric research nurses. The study comprises a total of 1655 live births. Offspring were followed up at birth 3 and 12 months. At the 3-month visits, a questionnaire on feeding practice, whether breast, formula milk, or mixed, was administered to the mothers. In September 2006, abdominal US was introduced to the follow-up protocol at ages 3 and 12 months and the current analysis is based on those infants with follow-up assessments between September 2006 and June 2010. In total, 487 infants (254 boys and 233 girls) had US measures at 3 months and 495 infants (258 boys and 237 girls) at 12 months. US measures at both 3 and 12 months were available in 360 infants (187 boys and 173 girls). Longitudinal data from birth were available on length, weight and skinfold thickness. No significant differences were observed between infants who had US only at 3 months, infants who had US only at 12 months, and those who had US at both 3 and 12 months with regard to gestational age, anthropometry at birth, and at 3 months (Table 1). Ethical approval was given by the Cambridge local research ethics committee and written informed consent was obtained from the mothers.

### 2.3. Anthropometry

In the validation study, weight, length, and waist circumference (WC) were measured by one of three trained clinical research fellows. Weight was measured using a Marsden Professional Baby Scale (London, UK) and recorded to the nearest 0.1 kg. Crown-heel length was measured with a Rollameter, a recumbent infant board with a sliding footboard (Raven Equipment Ltd., Dunmow, Essex, UK). WC was measured at the midpoint between the inferior border of the costal margin and the anterior superior iliac crests using a D-loop tape measure (Chasmors Ltd., London, UK) [26]. 

In CBGS, infants were measured at birth, 3 months, and 12 months by trained paediatric nurses or research assistants. Weight was measured to the nearest 1 g using a SECA 757 digital scale (Chasmors Ltd.) and length using a Kiddimeter (Chasmors Ltd). WC was measured as described previously. Triceps, quadriceps, flank, and subscapular skinfold thicknesses were measured in triplicate on the left side of the body using Holtain calipers (Chasmors Ltd). The triceps skinfold was measured halfway between the acromial process and the olecranon. The quadriceps skinfold was taken from a vertical line over the quadriceps muscle at midline of the thigh, and half way between the top of the patella and the inguinal crease. The flank (posterior suprailiac) skinfold was taken from the diagonal plane in line with the natural angle of the iliac crest taken in the posterior axillary line immediately posterior to the iliac crest. The skinfold was taken at the oblique angle below the left scapula [26]. Ponderal index was calculated as weight (kg)/length (m)^3^. SD scores (SDS) were derived for weight and length by comparison to the 1990 British reference [27]. Separate internal SDS were calculated for each skinfold thickness [=(individual measurement *minus* cohort mean)/cohort SD], and then the overall skinfold thickness SDS was calculated as the mean of the four skinfold SD scores in each individual. The relative intraobserver technical error of measurement (TEM) for length ranged between 0.03% and 0.05%, for quadriceps ranged between 0.4% and 0.6%, for triceps ranged between 1.9% and 2.4%, for subscapular ranged between 1.7% and 2.8% and for flank ranged between 0.8% and 2.0%. The relative interobserver TEM was 0.7% for length, 2.0% for quadriceps, 2.9% for subscapular, 2.2% for triceps, 3.2% for flank. The calculations were based on repeated measurements in 12 infants.

### 2.4. Ultrasound (US) Abdominal Depths

US-visceral depth and US-subcutaneous-abdominal depth were measured using a Logiq Book XP ultrasound, with a 3C MHZ -RS abdominal curved array transducer (both from GE Healthcare, Bedford, UK). For both measures, the transducer was positioned where the xiphoid line intercepted the WC measurement plane, and the images were taken during expiration. US-visceral depth was measured on a longitudinal plane with a probe depth of 9 cm and was defined as the distance between the peritoneal boundary and the corpus of the lumbar vertebra. US-subcutaneous abdominal depth was measured at the same location, but on a transverse plane with a probe depth of 4 cm, and was defined as the distance between the cutaneous boundary and the linea alba. The image was captured when the transducer just had contact with the skin to avoid compressing the subcutaneous adipose area. In the validation study, the US measures were performed by one of two trained operators and in CBGS by one of four trained operators. The relative intraobserver technical error of measurement (TEM) ranged between 0.3% and 1.7% for US-visceral depth, and 1.1% and 2.6% for US-subcutaneous-abdominal depth, and the relative interobserver TEM was 3.2% for US-visceral depth 3.6% for US-subcutaneous-abdominal depth, based on repeated measurements in 12 infants. In the validation study, qualitative information on the feasibility and acceptability of US was collected from the participants using open-ended questions. 

### 2.5. Magnetic Resonance Imaging (MRI)

The MRI procedure used in the validation study is described elsewhere [19]. Briefly, infants were scanned on the same day of the US measurements while in natural sleep, securely swaddled and wearing protective ear muffs, in a 1.5 T Philips Acheiva scanner (Best, Netherlands) using a rapid T1-weighted spin-echo sequence (repetition time 600 ms, echo time 16 ms, field of view =24 cm, number of signal averages =2, and a 256 × 256 matrix with phase conjugate symmetry). Five mm-thick contiguous transverse images throughout the body were obtained and were analysed using SliceOmatic (Tomovision, Montreal, QC, Canada), a semiautomated program containing a threshold range and a contour-following algorithm with an interactive slice editor facility to distinguish between adipose tissue compartments. IA-AT and SCA-AT volumes were calculated from the adipose tissue in the slices from the top of the sacrum to the slice containing the top of the liver or base of the lung [19]. Total body subcutaneous adipose tissue (total SC-AT) was also calculated and comprised both superficial and deep-subcutaneous adipose tissues [28]. 

### 2.6. Statistical Analyses

Statistical analyses were performed using STATA version 11.0 (StataCorp Ltd.). Means and standard deviations are presented separately for boys and girls and sex differences were tested using unpaired *t*-tests. For validation purposes, Pearson's correlation coefficients were used to describe the associations between IA-AT or SCA-AT and the US and anthropometric variables. Multiple regression was used to test the added contribution of US depths to anthropometry in explaining the variance in IA-AT or SCA-AT including the root mean square error (RMSE). 

For CBGS, Pearson's correlation coefficients were used to describe cross-sectional associations between US depths at 3 or 12 months and anthropometric variables. Associations between growth parameters at birth (birth weight and skinfolds SDS) and US depths at 3 or 12 months were tested using linear regression models. Associations were similar in both sexes, so all analyses were performed in the total sample with adjustment for sex. Further adjustment for current size (weight or skinfolds SDS) was included in the final models. Colinearity between parameters in the same model was quantified using the variance inflation factor (VIF); models with VIF > 5 were considered invalid [29]. To explore the strength of tracking in visceral and subcutaneous-abdominal depths, we performed Pearson's correlations in the 360 infants with US measures at both 3 and 12 months. Weak tracking was defined by a correlation coefficient <0.3, moderate tracking as 0.3–0.6, and strong tracking as >0.6 [30]. 

All body composition variables and the residuals of the regression models were normally distributed. Statistical significance was set at *P* < 0.05.

## 3. Results

### 3.1. Validation Study

In the 22 newborn infants, mean range for age was 10.6 (6–19) days; gestational age at birth 39.9 (37.1–40.8) weeks; weight 3.3 (2.5–3.9) kg; length 53.1 (47–57) cm; WC 34 (29–39) cm; IA-AT 18 (8–32) cm^3^, SCA-AT 104 (59–202) cm^3^; US-visceral depth 2.0 (1.2–3.0) cm; and US-subcutaneous abdominal depth 0.30 (0.2–0.4) cm. 

**Figure 1:**
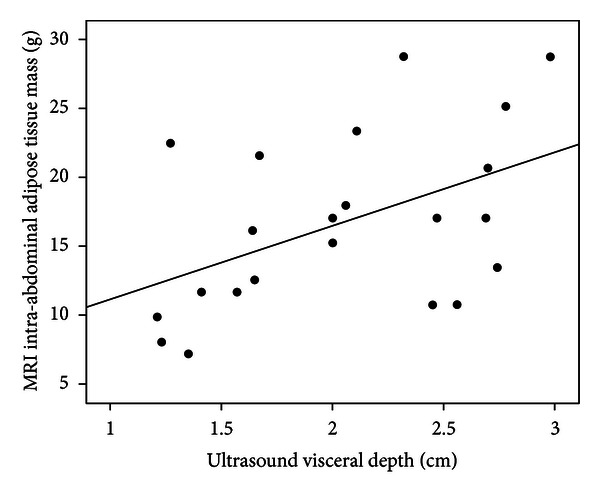
Scatterplot of ultrasound visceral depth against MRI intra-abdominal adipose tissue (IAT-AT) mass. Correlation coefficient: *r* = 0.48; *P* = 0.02.

**Figure 2:**
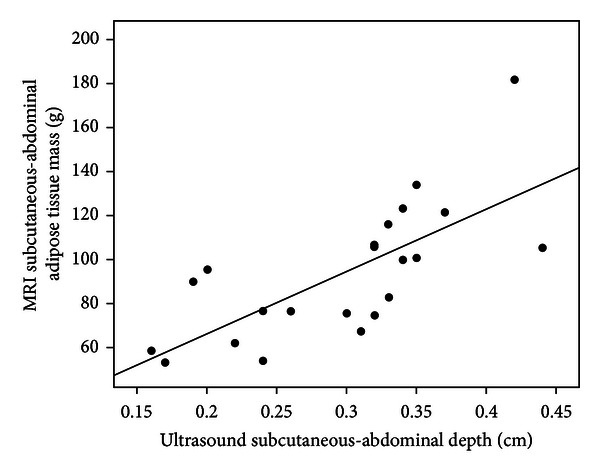
Scatterplot of ultrasound subcutaneous-abdominal depth against MRI subcutaneous-abdominal adipose tissue (SCAT-AT) mass. Correlation coefficient: *r* = 0.71; *P* < 0.001.

IA-AT showed moderate positive correlations with US-visceral depth (*r* = 0.48; *P* = 0.02) and US-subcutaneous abdominal depth (*r* = 0.52; *P* = 0.01), and these were higher than with any anthropometric variable (Table 2). SCA-AT was most strongly positively correlated with US-subcutaneous abdominal depth (*r* = 0.71; *P* = 0.002), followed by weight (*r* = 0.60; *P* = 0.003). US-subcutaneous abdominal depth was also strongly positively correlated with total SC-AT (*r* = 0.78; *P* < 0.0001), weight (*r* = 0.92; *P* < 0.0001), and length (*r* = 0.79; *P* < 0.0001). Examination of scatter plots (Figures 1 and 2) showed no obvious heteroscedasticity (i.e., the degree of scatter did not change with increasing IA-AT or SCA-AT). In the multiple regression models (Table 3), the addition of US-visceral depth to weight, sex, age, and US-subcutaneous abdominal depth improved the explained variance in IA-AT from 43% to 62% (*P* value for model change = 0.02). For the prediction of SCA-AT, the addition of US-subcutaneous abdominal depth to weight, sex and age improved the explained variance from 44% to 65% (*P* = 0.1). Accordingly, addition of the US parameters substantially reduced the root mean square error (RMSE) terms for SCAT-AT for IA-AT (Table 3).

Eleven mothers provided qualitative comments regarding the measurements. Nine mothers commented favourably on the shorter duration of US compared to MRI, and four commented favourably on the lack of separation from their infants using US. 

### 3.2. Abdominal Ultrasound in the Cambridge Baby Growth Study

Characteristics of CBGS infants with US measures at age 3 months (*N* = 487) or 12 months (*N* = 495) are summarised in Table 4. Boys had higher birth weights and birth lengths but lower skinfold thicknesses at birth compared to girls (*P* < 0.0001), despite no difference in gestational age (*P* > 0.05). Boys remained heavier and taller than girls at 3 and 12 months, and boys had slightly greater mean US-visceral depth than girls at 12 months (*P* = 0.04) but not at 3 months (*P* = 0.9). 

Mean US-visceral depth at age 12 months was 22% higher in boys and 17% higher in girls at 12 months than at 3 months. In contrast, mean US-subcutaneous abdominal depth and skinfold thickness did not change with age. The apparent increase in US-visceral depth was confirmed in the 360 infants with repeat measures at both 3 and 12 months (mean change: +0.4 cm; *P* < 0.0001). In this longitudinal sample US-visceral depth showed only weak tracking between 3 and 12 months (*r* = 0.11; *P* = 0.04). In contrast the inter-correlation coefficients between 3–12 months were stronger for mean skinfold thickness SDS (*r* = 0.30; *P* < 0.0001), ponderal index (*r* = 0.30; *P* < 0.0001), US-subcutaneous abdominal depth (*r* = 0.40; *P* < 0.0001), WC (*r* = 0.50; *P* < 0.0001), weight (*r* = 0.70; *P* < 0.0001), and length (*r* = 0.73; *P* < 0.0001). Despite these marked changes during infancy, US-visceral depths were consistently lower at both 3 and 12 months in infants who were exclusively breast-fed at age 3 months compared to other infants (at 3 months: mean ± SD: 2.3  ±  0.6 versus 2.4  ±  0.6 cm, *P* = 0.04; at 12 months: 2.7 ± 0.5 versus 2.8 ± 0.5 cm, *P* = 0.05). US-visceral depth was unrelated to time from last feed at 3 months (*r* = −0.01, *P* = 0.8) and 12 months (*r* = − 0.06, *P* = 0.1).

### 3.3. Abdominal Ultrasound Depth Related to Infancy Growth

In cross-sectional analyses (Table 5), US-visceral depth was positively associated with ponderal index at 3 months (*P* = 0.02) and with mean skinfold thickness SDS at 12 months (*P* = 0.02). In contrast, US-subcutaneous abdominal depth at both 3 and 12 months was positively associated with all measures of current body size (*P* < 0.005). 

In models without adjustment for current body size, US-visceral depth at 3 months (*P* = 0.06) and 12 months (*P* = 0.03) showed *inverse* trends or associations with skinfold thickness at birth, and these inverse associations strengthened on adjustment for current skinfold thickness (at 3 months: *P* = 0.03; at 12 months: *P* = 0.009) (Table 6). In contrast, US-subcutaneous abdominal depth at 3 months was *positively* associated with skinfold thickness at birth (*P* = 0.004), but not at age 12 months (*P* = 0.1) and no associations remained on adjustment for current skinfolds (Table 6). In unadjusted models no US measure was associated with birth weight; inverse associations between birth weight and US-subcutaneous abdominal depth at 3 and 12 months only emerged after adjustment for current body weight (*P* = 0.01 at both 3 and 12 months).

## 4. Discussion

Our validation study results showed that US abdominal depth provides acceptable accuracy in estimating IA-AT and SCA-AT volumes assessed by MRI in infants. The US measures showed stronger correlations with IA-AT and SCA-AT than did the traditional anthropometric variables, and the addition of US measures to those variables substantially improved the predictions of IA-AT and SCA-AT. The precision of our models was significantly improved as RMSE for IA-AT and SCA-AT substantially decreased. Furthermore, the reproducibility and reliability of the US measures were high as indicated by low inter- and intraobserver technical errors of measurement. In addition, the ultrasound method was highly acceptable to parents as it was faster to perform than MRI and no separation from their infants was required. By contrast, the actual MRI scanning time is approximately 12 minutes, but the whole procedure including preparation time to settle the infant can take up to one hour.

We acknowledge that our validation study has some limitations. In particular, it was performed in newborns at age range 6–19 days, rather than at 3 or 12 months as in CBGS. This is because the reference imaging techniques, MRI and CT, are not feasible for research studies at those later ages, as discussed previously. However, our findings are consistent with positive reports in adults and adolescents comparing abdominal US to MRI [20–23, 31, 32]. In contrast, our earlier validation study in young children aged 6-7 years old showed only weak correlations between US-measures and IA-AT, which was assessed in that study by single-slice CT at L4-L5 corresponding to the location of the US probe [33]. A few other studies have used a different US technique, the abdominal adipose tissue index, which is the ratio between the preperitoneal fat thickness and subcutaneous fat thickness [18, 34]. However, that technique has only been validated in adults [35, 36] and in one study of 34 children aged 1–18 years (only 9 were between 1 and 4 years old) [35, 36]. Further US validation studies are required in other childhood age groups using multiple slice assessment of IA-AT volumes as the reference.

Secondly, the sample size in our validation study was small (*n* = 22). In fact this study had 80% power to detect a Pearson's correlation coefficient higher than 0.56 with a type I error of 5%. Our inclusion criteria were limited to only healthy newborns (birth weight range 2.5–3.9 kg) due to the need to travel to a research site some miles from their place of birth. We anticipate that the inclusion of infants with more extremes of underweight/thinness and macrosomia would increase the strength of the observed correlations. We were unable to test absolute validity using the Bland-Altman analysis because this method requires the different measurements to be reported in the same units in order to calculate the degree of bias on the raw measurement scale. In addition, no existing prediction equations were available for IA-AT and SC-AT from US measures based on US measures in this age group. Future independent studies should test the absolute validity of the prediction models derived in this study. However, our main purpose was not to develop prediction models, but rather to analyze the associations between anthropometric variables, age, and gender with US parameters.

Finally, the correlation between US-visceral depth and IA-AT was only moderate (*r* = 0.48; *P* = 0.02). Indeed, US-subcutaneous abdominal depth showed a slightly stronger correlation with IA-AT (*r* = 0.52; *P* = 0.01), but was more strongly related to SCA-AT and hence US-visceral depth was the more specific marker of IA-AT. In contrast, the correlations between US-visceral depth and IA-AT were 0.80–0.82 in older adults and 0.64–0.72 in adolescents [20–24]. Lower IA-AT volumes in infants might contribute to these lower correlations. Also, in our experience measurement of US-visceral depth in infants is more susceptible to bowel peristalsis and movement artifacts than in older age groups; however US-visceral depth was unrelated to time from last feed. While more accurate markers would provide greater power for subsequent studies [37], such correlations are of similar strength as other proxy measures used in large epidemiological studies tools to assess physical activity and dietary behaviours. For example, questionnaire estimates of energy expenditure show correlations of 0.20 to 0.67 with the doubly labelled water reference techniques [38, 39], and questionnaire estimates of nutrient intakes show correlations of ~0.5 with nutritional biomarker references [40]. Therefore, we consider that US abdominal depth is suitable to rank infants with higher or lower abdominal adipose tissue volumes.

In the CBGS cohort study, we found that infants with lower skinfold thickness at birth tended to have *lower* subcutaneous abdominal depth at age 3 months, but *greater* visceral depths at ages 3 and 12 months, suggesting a differential regulation of these adipose tissue compartments. The stronger visceral depth associations that we observed with lower skinfold thickness at birth rather than lower birth weight suggest that these birth measures may be proxies for fetal growth restraint during the later antenatal period. In support of this notion, our previous studies using MRI in newborns reported that growth-restricted and extremely preterm infants have reduced SCAT but preserved IA-AT mass [19, 28]. Our findings of differential changes in visceral compared to subcutaneous abdominal depths with age and by sex further support the active partitioning of adipose tissue between these compartments during infancy.

We also observed that the associations between skinfold thickness at birth and infancy visceral depth strengthened with further adjustment for current skinfold thickness. Some investigators have argued that adjustment for current size could potentially introduce bias due to overcontrolling [41]. However, such adjustment can be justified if current body size is a potential confounder that is positively associated with both birth size and the outcome of interest. Our interpretation is in line with Lucas and colleagues [42], who have argued that if an association with birth size becomes apparent or is amplified after adjustment for current size, then it is the postnatal change in size between birth and followup that influences the outcome, rather than an antenatal factor.

Therefore, postnatal factors related to infancy gains in skinfold thickness may influence the accumulation of visceral adipose tissue at 3 and 12 months. Our observation of weak tracking in visceral depth indicates wide between-individuals variation in the rate of accumulation of visceral adipose tissue during infancy, although measurement error and imprecision are likely contributing factors to this estimate. Our observed associations with breastfeeding indicate that postnatal nutrition may influence the accumulation of visceral adipose tissue in infancy.

In conclusion, US abdominal depths were better than anthropometric measures in ranking infants with higher or lower IA-AT and SCA-AT volumes and may be applicable to large epidemiological studies at young ages when MRI and CT imaging techniques are infeasible. Application of these US measures in a large birth cohort study showed that visceral and subcutaneous-abdominal depths differed in their changes with age and in their patterns of association with antenatal and postnatal factors, suggesting that IA-AT and SCA-AT may be differentially regulated in the first year of life. 

## Figures and Tables

**Table 1:** Infants characteristics in the Cambridge Baby Growth Study with ultrasound measures at 3 months, 12 months and both at 3 and 12 months^1^.

	US at 3 months only		US at 12 months only		US at 3 and 12 months	
	Boys	Girls	Boys	Girls	Boys	Girls
	*n* = 67	*n* = 60	*n* = 108	*n* = 100	*n* = 187	*n* = 173
Birth						
Gestational age at birth (weeks)	39.6 ± 1.4	39.9 ± 1.2	39.8 ± 1.8	39.9 ± 1.1	39.8 ± 1.5	39.7 ± 1.6
Weight (kg)	3.5 ± 0.5	3.4 ± 0.5	3.5 ± 0.6	3.4 ± 0.4	3.5 ± 0.5	3.4 ± 0.5
Length (cm)	51.7 ± 2.4	51.0 ± 2.8	51.5 ± 2.2	51.0 ± 2.1	51.7 ± 2.8	51.7 ± 2.8
Ponderal index (kg/m^3^)	25.3 ± 3.2	26.0 ± 2.7	26.0 ± 3.6	26.0 ± 3.2	25.6 ± 3.2	25.9 ± 3.1
Sum of skinfolds (cm)	2.5 ± 0.6	2.5 ± 0.6	2.4 ± 0.5	2.5 ± 0.6	2.4 ± 0.6	2.5 ± 0.6

^1^Data are means (±standard deviations).

US: ultrasound.

**Table 2:** Validation study: intercorrelations between MRI IA-AT or SCA-AT and anthropometry or ultrasound measures in 22 term infants.

	IA-AT	SCA-AT	Total SC-AT	Ponderal Index	Length	Weight	US-SC-abdo depth	US-visceral depth
	(cm^3^)^1^	(cm^3^)^2^	(cm^3^)^3^	(kg/m^3^)	(cm)	(kg)	(cm)^4,5^
SCA-AT (cm^3^)^2^	0.48*	1						
Total SC-AT (cm^3^)^3^	0.61*	0.94**	1					
Ponderal Index (kg/m^3^)	0.15	0.32	0.27	1				
Length (cm)	0.34	0.40*	0.54*	−0.40*	1			
Weight (kg)	0.39	0.6*	0.70**	0.2	0.81**	1		
US-SC-abdo depth (cm)^4,5^	0.52*	0.71**	0.78**	0.17	0.79**	0.92**	1	
US-visceral depth (cm)^3^	0.48*	0.22	0.31	0.14	0.31	0.40*	0.38	1
Waist (cm)	0.08	0.16	0.26	0.19	0.54*	0.72**	0.6*	0.28

Values are Pearson's correlation coefficients.

*P value < 0.05; **P value < 0.001.

^1^IA-AT: internal-abdominal adipose tissue volume by MRI.

^2^SCA-AT: subcutaneous-abdominal adipose tissue volume by MRI.

^3^Total SC-AT: total body subcutaneous adipose tissue volume by MRI.

^4^US: Ultrasound.

^5^SC-abdo depth: subcutaneous-abdominal adipose tissue depth.

**Table 3:** Prediction models for IA-AT and SCA-AT in the validation study.

	Model^1^	Constant	*B* ^6^ ± SE					*R* ^2^ (%)	RMSE^7^	*P* value for model change
			Weight (kg)	Sex	Age (days)	US SC-abdo depth (cm)^4,5^	US-visceral depth (cm)^4^			
IA-AT (cm^3^)^2^	1	−1.4	5.9 ± 3.1	—	—	—	—	15	61.3	*0.07 *
	2	−1.5	5.7 ± 3.4	0.4 ± 3.1	—	—	—	16	59.8	*0.1 *
	3	−0.7	4.8 ± 3.4	−0.8 ± 3.2	0.3 ± 0.3	—	—	22	60.0	*0.2 *
	4	23.7	−12.9 ± 8.0	−0.3 ± 2.9	0.4 ± 0.3	113.8 ± 45.4	—	43	53.4	*0.1 *
	5	20.9	−15.0 ± 6.7	2.7 ± 2.6	0.5 ± 0.2	116.6 ± 38.1	6.6 ± 2.3	62	37.4	*0.02 *

SCA-AT (cm^3^)^3^	1	−42.6	43.6 ± 12.9	—	—	—	—	36	38.2	*0.003 *
	2	−48.6	36.2 ± 13.2	19.8 ± 12.2	—	—	—	44	37.4	*0.01 *
	3	−49.4	37.1 ± 14.0	21.0 ± 13.2	−0.3 ± 1.2	—	—	44	34.8	*0.02 *
	4	66.7	−47.4 ± 0.03	23.4 ± 0.01	0.08 ± 0.09	540.0 ± 171.4	—	65	20.2	*0.1 *

^1^Covariables were added sequentially to the prediction models to demonstrate their incremental benefits.

^2^IA-AT: internal-abdominal adipose tissue volume by MRI.

^3^SCA-AT: Subcutaneous abdominal adipose tissue volume by MRI.

^4^US: Ultrasound.

^5^SC-abdo: subcutaneous-abdominal.

^6^ *B*: regression coefficient (±respective standard error).

^7^RMSE: root mean square error.

^8^ *R* ^2^: coefficient of determination.

**Table 4:** Summary of measurements in Cambridge Baby Growth Study infants.

	Boys	Girls	*P* value^1^
Birth	*n* = 362	*n* = 333	
Gestational age at birth (weeks)	39.8 ± 1.6	39.9 ± 1.3	*0.6 *
Weight (kg)	3.5 ± 0.5	3.4 ± 0.4	*0.006 *
Length (cm)	51.5 ± 3.5	51.0 ± 2.6	*0.004 *
Ponderal index (kg/m^3^)	26.0 ± 3.4	26.0 ± 3.1	*0.2 *
Sum of skinfolds (cm)	2.4 ± 0.6	2.5 ± 0.6	*0.04 *

3 months^2^	*n* = 254	*n* = 233	
Weight (kg)	6.4 ± 0.83	5.8 ± 0.7	*<0.0001 *
Length (cm)	61.8 ± 2.5	60.2 ± 2.5	*<0.0001 *
Ponderal index (kg/m^3^)	27.0 ± 2.1	27.0 ± 2.4	*0.1 *
Sum of skinfolds (cm)	4.4 ± 0.8	4.4 ± 0.8	*0.6 *
US-visceral depth (cm)	2.3 ± 0.6	2.3 ± 0.6	*0.9 *
US-subcut abdo depth (cm)	0.4 ± 0.1	0.4 ± 0.1	*0.7 *

12 months^3^	*n* = 258	*n* = 237	
Weight (kg)	10.2 ± 1.1	9.6 ± −1.1	*<0.0001 *
Length (cm)	76.4 ± 2.7	74.9 ± 2.6	*<0.0001 *
Ponderal index (kg/m^3^)	23.0 ± 1.6	23.0 ± 1.8	*0.7 *
Sum of skinfolds (cm)	4.3 ± 0.8	4.5 ± 0.8	*0.01 *
US-visceral depth (cm)^4^	2.8 ± 0.6	2.7 ± 0.5	*0.04 *
US-subcut abdo depth (cm)^4^	0.4 ± 0.1	0.4 ± 0.1	*0.6 *

Data are means (±standard deviation).

^1^Student's t-test was used to compare boys versus girls.

^2^3-month ultrasound measurements were performed in 487 infants (254 boys and 233 girls).

^3^12-month ultrasound measurements were performed in 495 infants (258 boys and 237 girls).

^4^US: ultrasound.

**Table 5:** Cross-sectional correlations between anthropometry^1^ and abdominal ultrasound measures at 3 months (487 infants) and 12 months (495 infants). Data are Pearson's coefficients.

	US-visceral depth		US-subcutaneous abdominal depth	
	3 months	12 months	3 months	12 months
Anthropometry at 3 months				
Weight SDS	0.02		0.31**	
Length SDS	−0.05		0.20**	
Ponderal index SDS	0.11*		0.27**	
Mean of skinfolds SDS	0.05		0.31**	
Anthropometry at 12 months				
Weight SDS		0.03		0.30**
Length SDS		0.00		0.11**
Ponderal index SDS		0.04		0.26**
Mean of skinfolds SDS		0.10*		0.30**

^1^SDS: sex- and age-adjusted standard deviation scores.

^2^US: ultrasound.

*P < 0.05, **P < 0.005.

**Table 6:** Associations between size at birth and ultrasound abdominal depth measurements at 3 months (487 infants) and 12 months (495 infants).

	Birth weight SDS		Mean skinfold thickness SDS at birth	
	*B* ± SE^1^	P value	*B* ± SE^1^	P value
Model 1				

US-visceral depth (cm)				
3 months	−0.024 ± 0.027	*0.4 *	−0.059 ± 0.031	*0.06 *
12 months	−0.041 ± 0.024	*0.09 *	−**0.062** ± **0.028**	**0.03**
US-subcut abdo depth (cm)				
3 months	0.005 ± 0.005	*0.3 *	**0.015** ± **0.005**	**0.004**
12 months	0.002 ± 0.004	*0.6 *	0.007 ± 0.005	*0.1 *

Model 2				

US-visceral depth (cm)				
3 months	−0.041 ± 0.031	*0.2 *	−**0.073** ± **0.033**	**0.03**
12 months	−0.045 ± 0.026	*0.09 *	−**0.073** ± **0.028**	**0.009**
US-subcut abdo depth (cm)				
3 months	−**0.012** ± **0.005**	**0.01**	0.005 ± 0.005	*0.3 *
12 months	−**0.011** ± **0.004**	**0.01**	0.002 ± 0.005	*0.7 *

Results are shown before (Model 1) and after (Model 2) adjustment for body size at the time of the ultrasound measurement.

Model 1: adjusted for sex.

Model 2: also adjusted for current weight or skinfolds, respectively.

^1^ *B*: Regression coefficient (and respective standard error); this represents the SD change in each parameter per 1 SDS change in birth weight or skinfold thickness at birth.
